# Supplementary material for: Mechanisms governing the pioneering and redistribution capabilities of the non-classical pioneer PU.1
Source: Nat Commun. 2020 Jan 21;11:402. doi: 10.1038/s41467-019-13960-2 (PMC6972792; doi:10.1038/s41467-019-13960-2)
Supplement: Supplementary file 7 — Source data [file 41467_2019_13960_MOESM7_ESM.zip › Source_Data/FigureS3/FigureS3H_MotifScanOutput/homerResults/motif30.similar.html]

motif30

## Information for motif30

A
C
G
T
A
C
T
G
C
G
T
A
T
A
G
C
A
G
T
C
A
G
C
T
C
G
T
A
A
T
G
C
C
G
A
T
A
C
G
T
A
G
C
T
  
Reverse Opposite:  

T
C
G
A
C
G
T
A
C
G
T
A
T
A
C
G
A
C
G
T
C
T
G
A
C
T
A
G
A
T
C
G
A
C
G
T
A
G
T
C
C
G
T
A
  

|  |  |
| --- | --- |
| p-value: | 1e-27 |
| log p-value: | -6.221e+01 |
| Information Content per bp: | 1.886 |
| Number of Target Sequences with motif | 72.0 |
| Percentage of Target Sequences with motif | 1.86% |
| Number of Background Sequences with motif | 137.9 |
| Percentage of Background Sequences with motif | 0.31% |
| Average Position of motif in Targets | 230.4 +/- 180.9bp |
| Average Position of motif in Background | 197.7 +/- 125.5bp |
| Strand Bias (log2 ratio + to - strand density) | -0.4 |
| Multiplicity (# of sites on avg that occur together) | 1.03 |
| Motif File: | file (matrix) reverse opposite |

### Similar de novo motifs found

|  |  |  |  |  |  |  |  |
| --- | --- | --- | --- | --- | --- | --- | --- |
| Rank | Match Score | Redundant Motif | P-value | log P-value | % of Targets | % of Background | Motif file |
| 1 | 0.971 | A C G T A C T G C G T A A G T C A T G C A C G T C G T A G A T C C G A T A C G T | 1e-22 | -51.638847 | 1.94% | 0.42% | motif file (matrix) |
| 2 | 0.963 | A C T G G A C T C T A G T C G A A T G C G A T C C G A T T C G A T A G C G C A T A G C T G C A T | 1e-19 | -44.627566 | 2.97% | 1.04% | motif file (matrix) |
| 3 | 0.855 | A C G T C T A G C G T A A G C T A T G C A C G T C G T A G T A C C G A T | 1e-14 | -33.036323 | 1.32% | 0.31% | motif file (matrix) |
